# Supplementary material for: Mapping the evolution of fertility support policies in China: A content and instrumental analysis
Source: PLoS One. 2025 Oct 9;20(10):e0332137. doi: 10.1371/journal.pone.0332137 (PMC12510515; doi:10.1371/journal.pone.0332137)
Supplement: S1 Appendix — (ZIP) [file pone.0332137.s001.zip › S1 Appendix. 226 original policy documents/197-国家发展改革委、国家人口计生委关于印发“十二五”人口和计划生育服务体系建设规划的通知(FBM-CLI-4-192998).doc]

国家发展改革委、国家人口计生委关于印发“十二五”人口和计划生育服务体系建设规划的通知

**发布部门：** [国家发展和改革委员会(含原国家发展计划委员会、原国家计划委员会)](https://www.pkulaw.com/law/chl?Aggs.IssueDepartment=60301) [国家人口和计划生育委员会(含国家计划生育委员会)(已撤销)](https://www.pkulaw.com/law/chl?Aggs.IssueDepartment=60304) [机构沿革](javascript:void(0))

**发布日期：**2012.11.12

**实施日期：**2012.11.12

**时效性：** [现行有效](https://www.pkulaw.com/law/chl?Aggs.TimelinessDic=01)

**效力级别：** [部门工作文件](https://www.pkulaw.com/law/chl?Aggs.EffectivenessDic=XE0303)

**法规类别：** [人口计划管理](https://www.pkulaw.com/law/chl?Aggs.Category=09701)

国家发展改革委、国家人口计生委关于印发“十二五”人口和计划生育服务体系建设规划的通知

各省、自治区、直辖市及计划单列市、新疆生产建设兵团、黑龙江农垦总局发展改革委、人口计生委：
　　为贯彻落实《[国民经济和社会发展第十二个五年规划纲要](https://www.pkulaw.com/chl/603c7a7f2158b52bbdfb.html)》和《国家基本公共服务体系“十二五”规划》，国家发展改革委、国家人口计生委共同编制了《“十二五”人口和计划生育服务体系建设规划》（以下简称《规划》）。现印发给你们，请认真贯彻执行，并就有关事项通知如下：
　　一、《规划》由国家发展改革委和国家人口计生委共同编制和组织实施，规划期限为2012-2015年。请各省（区、市）按国家统一部署分年度组织执行。

　　二、中央专项支持各省（区、市）的项目数量在《规划》实施中进一步核定。国家发展改革委和国家人口计生委将根据各地经济发展水平、人口规模、计划生育服务机构设施设备状况以及有关政策因素等综合平衡，分省匡算，分年确定各省中央投资额度和支持项目数量。

　　三、请各地按照《[中央预算内投资补助和贴息项目管理暂行办法](https://www.pkulaw.com/chl/230c2ce6118e7be3bdfb.html)》（国家发改委第31号令）以及建设项目管理程序要求，认真做好项目前期准备工作。加强对《规划》实施的监督检查，适时开展评估工作。
　　附件：“十二五”人口和计划生育服务体系建设规划

国家发展改革委
国家人口计生委
2012年11月12日

　　附件：

“十二五”人口和计划生育服务体系建设规划

　　为坚定不移地落实计划生育基本国策，全面加强新时期人口和计划生育工作，稳定低生育水平，统筹解决人口问题，促进人口长期均衡发展，充分发挥人口和计划生育服务体系在人口和计划生育事业发展中的基础性、保障性作用，依据《[中华人民共和国国民经济和社会发展第十二个五年规划纲要](https://www.pkulaw.com/chl/603c7a7f2158b52bbdfb.html)》、《国家人口发展“十二五”规划》和《国家基本公共服务体系“十二五”规划》，制定本规划。

　　一、规划背景
　　人口和计划生育服务体系是直接为广大群众提供计划生育、优生优育、生殖健康服务的重要载体，是落实计划生育基本国策的物质基础和技术条件，是我国基本公共服务体系的重要组成部分，主要承担宣传教育、技术服务、优生指导、药具发放、信息咨询、随访服务、生殖健康和人员培训等八项任务。
　　“十一五”期间，为提升农村计划生育服务能力，国家发展改革委和国家人口计生委组织实施了《“十一五”期间农村基层计划生育服务体系建设规划》，有力改善了农村基层计划生育服务设施条件，技术服务能力有了较大提高，对于全面落实计划生育基本国策，稳定低生育水平，保障人民群众生殖健康权益，发挥了重要作用。
　　“十二五”是我国人口和计划生育事业发展的关键时期，也是人口和计划生育服务体系稳步提升、再上水平的重要阶段。经过长期不懈的艰苦努力，我国已实现了人口再生产类型的历史性转变，人口过快增长的势头得到有效遏制，增速逐步趋缓。但是，我国人口众多的基本国情没有改变，经济社会发展和资源环境长期面临较大人口压力的总体形势没有改变，落实计划生育基本国策、统筹解决人口问题和促进人口长期均衡发展的根本要求没有改变。未来一段时期，人口和计划生育服务将面临新形势、新任务和新要求。一是服务需求持续增长。我国总人口继续保持增长态势，育龄妇女数量居高不下，流动人口仍将保持庞大规模，出生缺陷发生率和出生性别比持续偏高，客观上要求增加服务供给。二是服务任务更加繁重。服务内容由基本四项服务向出生缺陷预防、信息咨询、育龄妇女随访及生殖健康普查等综合服务发展，国家免费孕前优生健康检查的全面开展，都将进一步增加工作量。三是服务要求明显提高。随着以人为本理念日益深入人心，需要不断改进服务方式和技术手段，提供更为高效、便捷、优质的服务。因此，必须高度重视人口和计划生育服务体系建设，不断加强基层和基础工作，筑牢人口和计划生育事业发展基础。
　　目前，人口和计划生育服务体系发展还不完善，难以满足人民群众日益增长的服务需求，主要表现在：一是全国农村基层尚有部分县级服务站未达到《农村计划生育服务机构基础设施建设标准》（建标[2005]206号）要求，其中85%以上分布在中西部地区，开展标准化、规范化服务难度较大；二是县级服务机构现有设备老化和不足并存，调查显示开展国家免费孕前优生健康检查服务所需的全自动生化分析仪、化学发光免疫分析仪、酶标分析仪等主要设备缺口都在50%以上，制约了服务开展和质量提升；三是“九五”和“十五”期间配备的计划生育流动服务车都将于“十二五”期间达到报废年限或里程，影响流动服务的有效开展；四是人口和计划生育服务体系的科技支撑和技术指导较为薄弱，现有计划生育科研院所基础设施老化、科研设备陈旧，研发和指导作用难以有效发挥。因此，通过统筹规划，进一步健全人口和计划生育服务体系，加强薄弱环节建设十分必要。

　　二、基本原则
　　以科学发展观为指导，以提高服务能力、服务质量为目标，全面加强人口和计划生育服务机构建设，健全服务体系，完善服务功能，丰富服务内涵，提高管理水平，打造专业化的服务队伍，开展群众满意的优质服务，为稳定低生育水平、促进人口长期均衡发展提供保障。
　　（一）统筹发展，全面推进
　　科学规划，逐步缩小地区之间、城乡之间的差距，努力实现计划生育基本公共服务均等化。分类指导，加大对中西部贫困地区、革命老区、民族地区和边疆地区人口和计划生育服务体系的支持力度。
　　（二）需求导向，突出重点
　　适应新形势、新要求，以服务需求为中心，以群众满意为标准，重点加强县级基础设施建设，扎实提高基层技术服务水平。着力完善科技支撑体系，努力提升计划生育科技创新和服务能力。
　　（三）地方主导，中央扶持
　　地方各级政府要按照中央统一部署，有步骤、分年度推进人口和计划生育服务体系建设，在规划布局、建设投入、用地安排和运行保障等方面负起主要责任。中央予以适当支持和补助，加强全过程监督管理和检查评估。
　　（四）健全体系，完善功能
　　围绕计划生育、优生优育、生殖健康等服务需求，构建以县乡服务机构为依托，流动服务车为纽带，功能齐全、布局科学、技术先进、服务优良，多层次的计划生育服务网络。按照“属地化管理、市民化服务”的原则，将流动人口计划生育管理服务纳入经常性工作范围，提供与户籍人口同等的计划生育基本公共服务。
　　（五）规范建设，高效运行
　　坚持标准化、规范化发展方向，合理控制建设规模，加强建设项目的全程管理和监督。坚持改革创新，加强人才队伍建设，完善服务保障机制，提高服务效率和服务水平，确保服务机构良性运行和可持续发展。

　　三、目标任务
　　通过深化改革，加大投入，健全体系，科学管理，全面实现计划生育服务设施标准化、服务管理规范化、服务队伍职业化、服务保障制度化，为落实计划生育基本国策、促进人口长期均衡发展奠定坚实基础。
　　--基本实现县县都有标准化的计划生育服务设施。根据实际需要，按照《农村计划生育服务机构基础设施建设标准》，对未达标的县级服务站（含少量市辖区未设立服务站的地市级服务站）进行标准化、规范化改扩建，计划建设规模约96万平方米。
　　--基本配齐县级服务站孕前优生健康检查设备。按照填平补齐的原则，为县级服务站（含少量市辖区未设立服务站的地市级服务站）配置约1万台检测设备，逐步满足开展免费孕前优生健康检查需求。
　　--继续实施计划生育流动服务车项目。制定服务车配备标准，为部分县级服务站配置流动服务车，保证计划生育流动服务不断档。建立车辆更新机制，完善运行保障制度。
　　--支持人口和计划生育服务体系科技支撑平台建设。围绕出生缺陷预防、生育调控技术、生殖与遗传等重点研发领域，依托现有省级计划生育科研院所，重点支持部分省级人口计划生育研发和指导中心的基础设施改造和设备升级，提升科技创新和服务能力，构建人口和计划生育服务体系科技支撑平台。

　　四、资金筹措
　　地方各级政府是人口和计划生育服务体系建设的责任主体，要切实履行基本公共服务职能，强化支出责任，加大资金投入，安排财政性专项资金，支持人口和计划生育服务体系建设。
　　中央安排预算内投资对人口和计划生育服务体系建设予以专项补助，根据财力情况，逐年实施。依据《农村计划生育服务机构基础设施建设标准》，对西部地区、中东部享受西部政策地区的基础设施建设和设备购置项目，中央补助不高于总投资的80％；对中部地区其他县（市）、东部地区享受中部政策地区，中央补助不高于总投资的60％；对东部地区其他县（市），中央补助不高于总投资的25％。对西藏、新疆南疆三地州和四省藏区等特殊困难地区，由中央根据实际情况予以全额补助。

　　五、保障措施
　　（一）加强组织领导
　　各地要从落实计划生育基本国策、统筹解决人口问题和实现人口长期均衡发展的高度来认识人口和计划生育服务体系建设的重要性和紧迫性，将其摆上重要议事日程，纳入经济社会发展规划，切实抓实抓好。要建立由发展改革、人口计划生育部门牵头，相关部门参与的工作机制，加强组织领导和沟通协调，做好规划组织实施工作，确保规划目标如期实现。
　　（二）完善运行机制
　　原则上，中央补助购置的孕前优生健康检查设备和流动服务车为一次性安排，地方各级政府要创造条件，多方筹集资金，加快建立运行保障和维护更新机制，确保设备和车辆的有效运行和及时更新。运行保障和维护更新机制要与各地人口和计划生育服务体系建设方案同步规划、同步审核、同步检查。
　　（三）加大资金投入
　　各地要按照分级负担、共同筹措的原则，切实加大投入力度，落实建设资金，确保足额及时到位、不留缺口。配套资金不落实的项目，不得申请中央补助投资。地方项目建设所需资金不得挤占计划生育事业费。积极支持创建幸福家庭活动，优先安排幸福家庭试点单位设备配备。
　　（四）严格项目管理
　　遵循基本建设程序，严格执行目标责任制、项目法人制、招标投标制、工程监理制。切实加强投资管理，厉行节约，合理控制设备采购种类和档次以及服务机构建设规模。项目建设资金应实行专户管理，封闭运行。严禁挪用、截留、挤占项目建设资金。加强服务机构的运行监管和设备的使用管理，健全制度，落实责任。
　　（五）加快人才培养
　　制定各级服务机构人员配置标准。加强教育培训，推进在职学历教育，提高服务人员素质，建设一支专业化的计划生育服务队伍。完善专业技术人员职称制度。认真贯彻落实《[关于加强社会工作专业人才队伍建设的意见](https://www.pkulaw.com/chl/3e711898022de366bdfb.html)》（中组发〔2011〕25号），加快生殖健康咨询师培训和职业技能鉴定。逐步建立职业标准、学科教材、教育培训、评估认证和职业监管体系。
　　（六）深化体制改革
　　建立分级负责、协作配合、权责统一、运转有效的服务机构管理体制，强化县乡（镇）两级服务机构协调配合、共同发展的管理模式。在试点基础上，逐步推行乡站县管，统筹配置和利用计划生育服务资源，提高服务效率和服务质量。加强基层公共服务资源整合，充分利用乡镇卫生院、村卫生室等开展计划生育服务，努力实现共建共享。加强计划生育信息系统建设，提高管理效率和服务质量。
　　地方各级发展改革和人口计生部门应根据本规划，编制地方建设方案。

© 北大法宝：（[www.pkulaw.com](https://www.pkulaw.com/)) 专业提供法律信息、法学知识和法律软件领域各类解决方案。北大法宝为您提供丰富的参考资料，正式引用法规条文时请与标准文本核对。
欢迎查看所有[产品和服务](https://www.pkulaw.com/helps/2.html)。法宝快讯：[如何快速找到您需要的检索结果？](https://www.pkulaw.com/helps/70.html)[法宝](https://www.pkulaw.com/helps/69.html)V6有何新特色？

扫描二维码阅读原文

**原文链接：**<https://www.pkulaw.com/chl/d6b69b89acdeb6e9bdfb.html>
